# Osteopenia and Sarcopenia as Potential Risk Factors for Surgical Site Infection after Posterior Lumbar Fusion: A Retrospective Study

**DOI:** 10.3390/microorganisms10101905

**Published:** 2022-09-26

**Authors:** Alberto Ruffilli, Marco Manzetti, Tosca Cerasoli, Francesca Barile, Giovanni Viroli, Matteo Traversari, Francesca Salamanna, Milena Fini, Cesare Faldini

**Affiliations:** 1 1st Orthopaedic and Traumatologic Clinic, IRCCS Istituto Ortopedico Rizzoli, 40136 Bologna, Italy; 2 Complex Structure Surgical Sciences and Technologies, IRCCS Istituto Ortopedico Rizzoli, 40136 Bologna, Italy; 3 Scientific Direction, IRCCS Istituto Ortopedico Rizzoli, 40136 Bologna, Italy

**Keywords:** osteopenia, sarcopenia, risk factors, surgical site infection, lumbar spinal fusion

## Abstract

Surgical site infection (SSI) is a feared complication in spinal surgery, that leads to lower outcomes and increased healthcare costs. Among its risk factors, sarcopenia and osteopenia have recently attracted particular interest. The purpose of this article is to evaluate the influence of sarcopenia and osteopenia on the postoperative infection rate in patients treated with posterior fusion for degenerative diseases of the lumbar spine. This retrospective study included data from 308 patients. Charts were reviewed and central sarcopenia and osteopenia were evaluated through magnetic resonance images (MRI), measuring the psoas to lumbar vertebral index (PLVI) and the M score. Multivariate linear regression was performed to identify independent risk factors for infection. The postoperative SSI rate was 8.4%. Patients with low PLVI scores were not more likely to experience postoperative SSI (*p* = 0.68), while low M-score patients were at higher risk of developing SSI (*p* = 0.04). However, they did not generally show low PLVI values (*p* = 0.5) and were homogeneously distributed between low and high PLVI (*p* = 0.6). Multivariate analysis confirmed a low M score to be an independent risk factor for SSI (*p* = 0.01). Our results suggest that osteopenia could have significant impact on spinal surgery, and prospective studies are needed to better investigate its role.

## 1. Introduction

Degenerative disease of the lumbosacral spine is a frequent cause of low back pain and functional disability, often requiring surgical intervention [1,2]. Surgical Site Infection (SSI) is a very frequent complication after spinal surgery, leading to a significant worsening of outcome and increases in morbidity and healthcare costs [1,2]. For these reasons, interest has grown in identifying risk factors associated with SSI. Koutsoumbelis et al. [3] studied a large cohort of patients and showed that the risk of postoperative infection can be increased by surgical and patient-related factors [3]. The surgical factors identified include a crowded operating theatre (>10 people), longer operative time, higher intraoperative blood loss, and incidental durotomy [1,2,3,4]. Patient-related risk factors include older age, more comorbidities, smoking, preoperative hospitalization >1 week, chronic opioid use, and steroid use [5,6,7]. However, with an increasing elderly population, specific factors should be considered in these patients to optimize surgical outcomes. Among risk factors, sarcopenia and osteopenia have recently attracted interest as part of the so-called “fragility syndrome” [8]. Frailty has been strongly linked to postoperative complications and mortality; its prevalence has been described in many studies and varies widely. The largest cohort (53,080 patients undergoing a variety of spinal procedures) was analyzed by Flexman et al. [9]: frailty was present in 4% of the total population and in 8% of patients older than 65 years. However, this percentage further increased in other cohorts, rising to 59% in adult deformity surgery [10] and 83% in patients with metastatic spine tumors [11].

**Figure 1:**
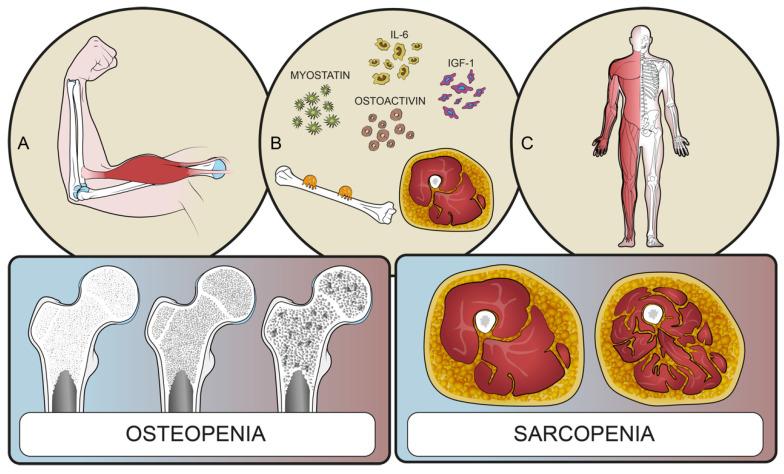
Interactions between bone and muscle tissues. (**A**) Muscles and bones interact with each other to ensure movement; (**B**) bone and muscles share common pathways involving inflammatory cytokines and catabolic metabolites; (**C**) muscles line the bones constituting a functional unit. These interactions could explain the common pathogenesis of sarcopenia, osteopenia, and frailty syndrome.

Central sarcopenia, defined as a “syndrome of progressive and generalized loss of muscle mass and strength”, is related to higher complication rates, longer length of stay, and higher peri-operative morbidity and mortality, in both spinal and prosthetic surgery [12,13,14]. Osteopenia, defined as decreased bone mineral density and bone mass, and measured by T score or Z score [15], is associated with vertebral fractures [16] and post-operative mechanical complications [8,16,17]. Moreover, sarcopenia and osteopenia are closely related to each other. In particular, the presence of reduced muscle mass is directly related to low bone density [8,18,19,20,21,22]. This relation has been explained by demonstrating that bone and muscle tissue interact reciprocally, acting as “endocrine targets” that communicate through paracrine and endocrine substances, modulating their development and function throughout the whole life of the patient [23,24,25] (Figure 1).

Common pathways involving inflammatory cytokines and anabolic and catabolic metabolites, and the mechanical interaction ongoing during physical activity, may contribute to the loss of muscle and bone mass. Preclinical and clinical data indicate the presence of many muscle-specific tissue factors that modulate bone tissue, such as insuline-like growth factor (IGF)-1, fibroblast growth factor (FGF-2), interleukin (IL)-6, IL-15, myostatin, osteoglycine, irisin, and ostoactivin [25,26]. The aforementioned factors contribute to and are involved in the pathogenesis of sarcopenia, and are also regulators of bone remodeling and therefore relevant to the reduction of bone mineral density [24]. Furthermore, osteopenia and sarcopenia are interrelated not only through these common molecular pathways involving cytokines and metabolites, but also through the mechanical interaction generated during physical activity.

To the best of the authors’ knowledge, there are currently no studies that correlate sarcopenia and osteopenia with rates of SSI after lumbar arthrodesis. Thus, the aim of the present study was to evaluate the influence of sarcopenia and osteopenia on the postoperative infection rate in a cohort of patients treated with posterior fusion for degenerative diseases of the lumbar spine.

## 2. Materials and Methods

### 2.1. Study Sample

After institutional review board approval (CE AVEC 208/2022/Oss/IOR), a retrospective review was performed focusing on patients aged 50 to 85 with degenerative lumbar spine disease treated with short posterior arthrodesis (3 levels or less) in our institution over a 15-year period (2005–2020).

Patients with a history of traumatic or neoplastic spine diseases, those who had already undergone spinal surgery in the past, and those with degenerative or idiopathic scoliosis were excluded. Patients without available imaging or who did not complete a minimum 2-year follow-up were also excluded.

### 2.2. Data Collection

Medical charts of the included patients were obtained and analyzed. Demographic data, age, gender, smoking history, Charlson Comorbidity Index (CCI), American Society of Anesthesiology (ASA) score, body mass index (BMI), and length of stay were collected. Postoperative infectious complications were recorded. Diagnosis of SSI was made by an infectious disease specialist based on clinical data, radiographic findings, blood tests, and/or documented positive culture obtained at the time of revision or debridement surgery, up to 2 years from the primary procedure.

Through magnetic resonance images (MRI), central sarcopenia and osteopenia were evaluated for every patient, measuring the psoas to lumbar vertebral index (PLVI) [27] and the M score [28], respectively.

**Figure 2:**
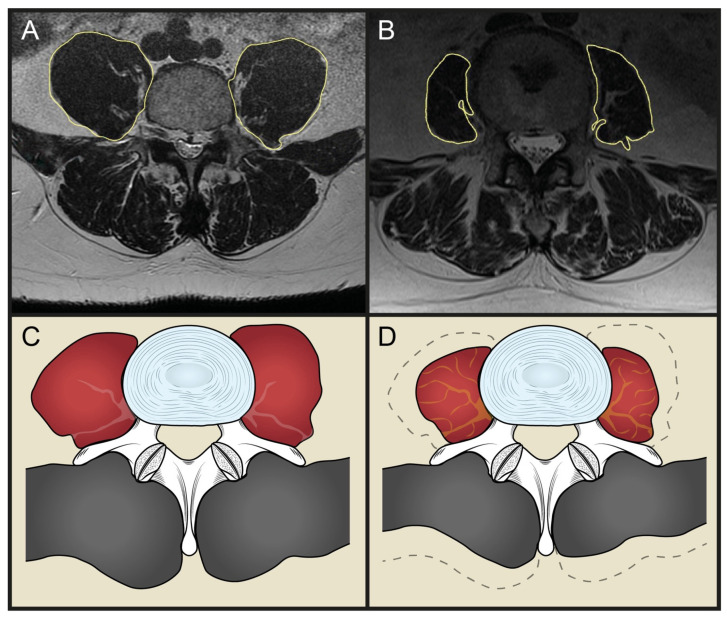
Examples of high (left) and low (right) PLVIs. (**A**) Example of high PLVI values in an MRI of the spine in axial view; (**B**) example of low PLVI values in an MRI of the spine in axial view; (**C**) example drawing of a non-sarcopenic psoas muscle (high PLVI); (**D**) example drawing of a sarcopenic psoas muscle (low PLVI).

The PLVI, a recently validated index of central sarcopenia [27], was measured by dividing the average cross-sectional area (CSA) of the psoas muscle by the average area of the L4 vertebrae, as described in previous studies [14,27]: PLVI = (left psoas CSA + right psoas CSA)\2\L4 vertebral body CSA (Figure 2).

The CSA values were measured on a single axial plane cut at the level of L4 pedicles.

**Figure 3:**
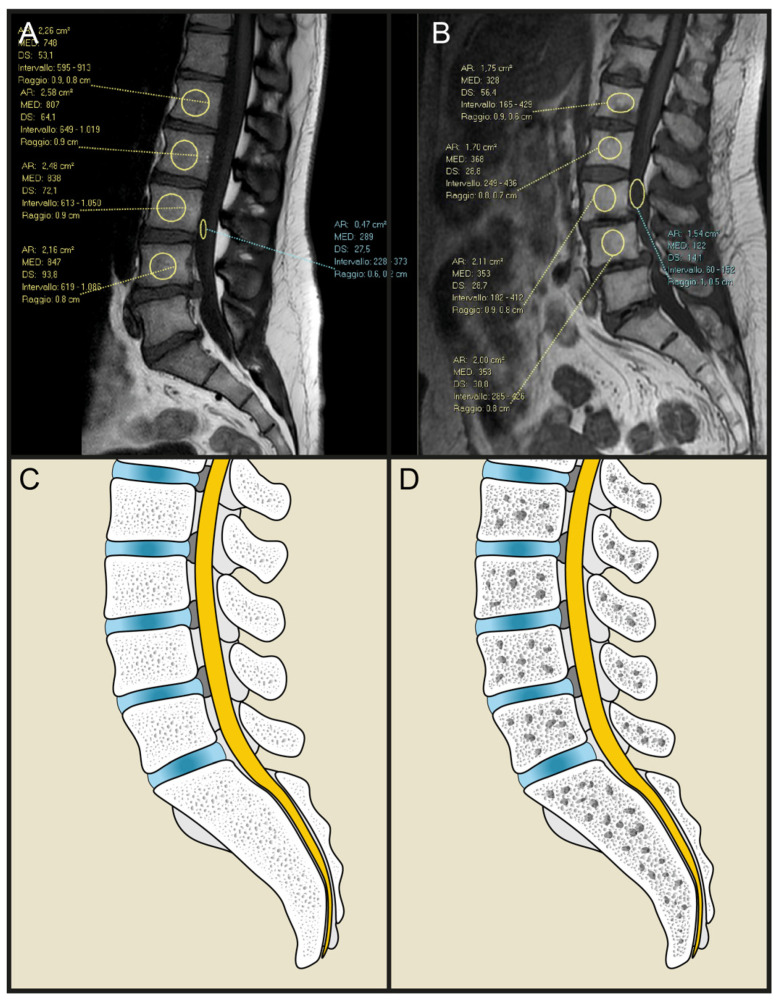
Examples of a high (left) and low (right) M scores. (**A**) Example of high M-score values in an MRI of the spine in sagittal view; (**B**) example of low M-score values in an MRI of the spine in sagittal view; (**C**) example drawing of a non-osteopenic spine (high M score); (**D**) example drawing of an osteopenic spine (low M score).

M score is a recently described [28] quantitative score of bone mass density. Routine lumbar spine MRIs was used to evaluate sagittal T1W spin-echo sequencing, appropriate for bone marrow assessment. A region of interest (ROI) (TR = 7, TE = 400–600, slice thickness = 4 mm, fov = 280 mm, matrix = 320 × 320), was applied manually as a circle in the vertebral bodies, from L1 to L4, excluding abnormalities, pathology, focal hemangiomas, and possible venous plexus. A ROI was also placed outside the patient to measure the noise, and signal-to-noise ratio (SNR_L1–L4_) was obtained by dividing the intravertebral intensity by the standard deviation of the noise. The mean (SNR_L1–L4_) and standard deviation (SD_ref_) of the reference population were then used and the M score was obtained according to the formula: M score = (SNR_L1–L4_ − SNR_ref_)\SD _ref_ (Figure 3).

All measurements were taken independently by two authors (MM, TC), both blinded to the other’s measurements and to the patient’s name. After checking for data accuracy and inconsistent results, the averages of the two authors’ measurements were recorded.

Patients were initially stratified into high and low PLVI groups, with the mean value (0.71) to identify the baseline, and then into quartiles; the same stratification was performed for M-score values, with the mean value 0 as baseline.

In addition, another statistical analysis was performed to stratify patients according to their postoperative status: infectious vs. non-infectious.

### 2.3. Statistical Analysis

Parametric testing was carried out to compare samples in terms of continuous variables and normal distribution. The Shapiro–Wilk test was applied to verify normal distribution. The Levene test was employed to analyze homogeneity of the variances. For the parametric test, we used the 2-tailed Student’s *t*-test to compare the average of the variables for homoscedastic unpaired groups, and the Welch *t*-test for non-homoscedastic unpaired groups. For the nonparametric test, we used the 2-tailed Mann–Whitney U test for unpaired groups.

“Post hoc” power analysis was not performed, because it has recently been declared an improper statistical tool for use in retrospective studies, and has been used to discredit the non-significance of evidence obtained [29].

Multivariate linear regression was performed to identify independent risk factors for infection.

*p* values < 0.05 were considered significant. All statistical analyses were performed using the Statistical Package for Social Science (IBM SPSS Statistics for Windows, Version 26.0; IBM Corp., Armonk, NY, USA).

## 3. Results

### 3.1. Demographic Data

A total of 308 patients (148 males, 48%, and 160 females, 52%) met the inclusion criteria. Mean age at surgery was 63.8 (range 51 to 82) and mean follow-up was 45.6 (range 24 to 124). Mean PLVI was 0.71 (range 0.18 to 1.54) and mean M score was 0 (range −1.74 to + 3.18). Baseline characteristics are summarized in Table 1.

Postoperative surgical site infection (SSI) was diagnosed in 26/308 patients (8.4%), at an average time of 30 days after surgery (range 14 to 43).

The responsible bacteria were Methicillin-susceptible *Staphylococcus aureus* in twelve cases (46.1%), Methicillin-resistant *S. aureus* in six cases (23.1%), *Enterobacter cloacae* in four cases (15.4%) and *Escherichia coli* in four cases (15.4%).

### 3.2. High vs. Low PLVI Patients

Of the included patients, 153 had low PLVI (LPLVIs) and 155 had high PLVI (HPLVIs). The two groups were significantly different in some of their baseline characteristics. Low PVI patients were more frequently older (65.3 ± 6.3 vs. 62.3 ± 5.6, *p* < 0.01), female (112/153 vs. 48/155, *p* = 0.016), with a smoking history (46/153 vs. 28/155, *p* = 0.016), and a higher Charlson Comorbidity Index (CCI, 2.7 ± 1.6 vs. 2.3 ± 1.02 *p* = 0.015). Moreover, LPLVIs had longer lengths of stay (12.2 ± 17.1 vs. 10.1 ± 5.5, *p* = 0.57) and operative times (185.1 ± 62.5 min vs. 197.41 ± 57.3 min, *p* = 0.25). However, low PLVI patients were not more likely to experience postoperative SSI (14/138 vs. 12/140, *p* = 0.68).

### 3.3. High vs. Low M-Score Patients

High and low M-score patients were significantly different in the following characteristics: low M-score patients were older (64 ± 6.4 vs. 62.3 ± 5.36; *p* = 0.05), more often had diabetes (68/166 vs. 2/20, *p* = 0.04), with higher ASA scores (2.06 ± 0.58 vs. 1.91 ± 0.61, *p* = 0.07), and BMI (26.7 ± 3.7 vs. 25.8 ± 3.5, *p* = 0.07).

Moreover, low M-score patients were at higher risk of developing SSI (10/95 vs. 1/35; *p* = 0.04). However, they did not show lower PLVI values (0.72 ± 0.2 vs. 0.70 ± 0.2, *p* = 0.5) and were homogeneously distributed between low and high PLVI (34/98 vs. 36/90; *p* = 0.6).

### 3.4. Infectious Status

The postoperative SSI rate in our cohort was 8.4% (26/308). When stratifying for postoperative SSI state, some baseline characteristics showed statistically significant differences: patients in the infected group were older (65.9 ± 7.9 vs. 63.6 ± 5.9, *p* = 0.002) and had higher CCI scores (3.4 ± 1.9 vs. 2.49 ± 1.35, *p* < 0.001).

However, the infected group did not differ in terms of average PLVI (0.75 ± 0.2 vs. 0.71 ± 0.19, *p* = 0.24) or M score (0.03 ± 1.02 vs. −0.20 ± 0.62, *p* = 0.29) when compared with the noninfected group. Therefore, while increasing age and higher CCI acted as risk predictors of postoperative SSI, the patients’ PLVI and M scores did not.

### 3.5. Multivariate Analysis

**Figure 4:**
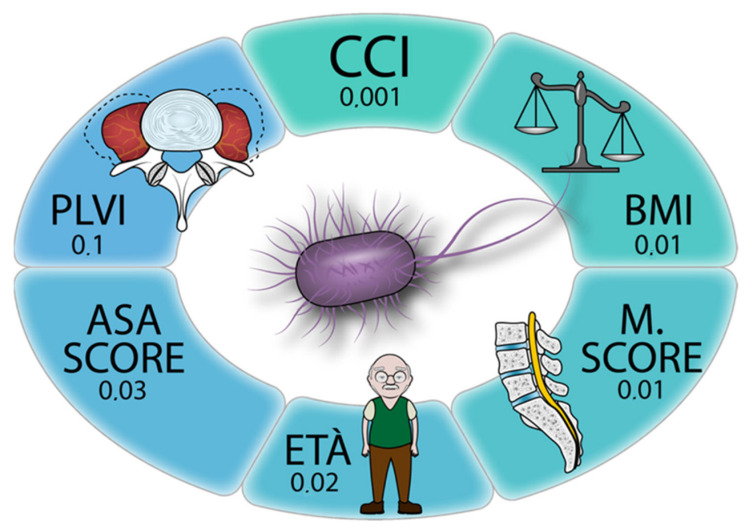
Multivariate linear regression analysis results.

Multivariate linear regression (Table 2 and Figure 4) confirmed that M score was an independent risk factor for infection (*p* = 0.01), as were length of stay (*p* < 0.001), age at surgery (*p* = 0.02), CCI (*p* = 0.001), and ASA score (*p* = 0.03).

## 4. Discussion

With an incidence that ranges from 0.2% to 16%, surgical site infection (SSI) is considered the third most common complication following spinal surgery [1,2,30].

Its treatment can require prolonged antibiotic therapy, multiple revision surgeries, long hospitalization, and even advanced soft-tissue reconstruction [1,2,19]. Therefore, understanding specific risk factors for SSI following spinal surgery is of paramount importance for surgeons and patients.

In this study we evaluated whether sarcopenia and osteopenia are risk factors for postoperative infection after short lumbar spinal fusion for degenerative diseases. Our findings were twofold: first, sarcopenia (low PLVI) and osteopenia (low M score) were not correlated with each other; second, while a low M score correlated with an increased risk of SSI, a low PLVI did not. The first finding is surprising. The interaction between bone and muscle tissue has been widely demonstrated, and some authors have hypothesized that sarcopenia and osteopenia share the same pathophysiology, through common molecular factors, hormonal imbalances, and increased cytokine activity, leading to physical decay [23,24,25,26]. A possible explanation for this result is that only PLVI and M score were used to define central sarcopenia and osteopenia, respectively. Moreover, PLVI is a measure of volume while M score is a measure of bone mass density; therefore, their trends can be difficult to compare.

Regarding the second finding, while the relationship between osteopenia and mechanical complications after lumbar arthrodesis has been widely described [8,16,17], currently no studies are available that analyze the incidence of postoperative SSI in relation to M-score values. However, our results are in line with those of other authors who identified the presence of osteoporosis (diagnosed with preoperative DEXA) as a risk factor for SSI. Lai et al. [31] conducted a retrospective multivariate analysis to analyze the risk factors for acute SSI following lumbar surgery, and found that patients with osteoporosis had an increased risk of postoperative infection. Similarly, Koutsoumbelis et al. [3] examined the medical records of 3218 patients who underwent posterior lumbar fixation, and identified those who developed postoperative infection: osteoporosis was significantly associated with postoperative infection in the results of the multivariate analysis. However, both of these studies included different surgical procedures (anterior, posterior, or combinate approaches) for several diseases (scoliosis, fractures, tumors); moreover, the presence of osteoporosis was not quantified, but only presented as categorical data (yes/no).

Considering PLVI, our results stand in contrast with those of other authors, who described sarcopenia as a risk factor for postoperative SSI [32,33]. Bokshan et al. [32] evaluated 46 patients and found that sarcopenic patients had a threefold increase in perioperative complications after thoracolumbar surgery. Similarly, Zakaria et al. [33] evaluated 395 patients undergoing posterior lumbar fusion, finding that those with lower psoas muscle area had increased risk of postoperative complications (including SSI). However, SSI was not the only focus of these studies; any kind of severe postoperative complication was considered. Patients were not stratified by indication (degenerative, infection, tumor, or trauma) or surgical procedure (any lumbar spine surgery was included, such as multilevel operations and/or revision surgery). Another important difference between the present research and the previously cited studies was the choice of limited age range (50–85), which helped to identify sarcopenia and osteopenia as pathological entities separate from the physiological mass loss in muscle and bone associated with senescence. Zakaria et al. [33] included patients of any age, with an average age similar to our patients (63.3 years) but an extremely high SD (±12.48, range 23–88 years). Bokshan et al. [32] included any patient older than 55 years, obtaining a high difference in average age between sarcopenic and nonsarcopenic groups (76.4 vs. 69.9 years).

Regarding our results for baseline characteristics, not surprisingly, age was the only factor related to low PLVI and M-score values as well as infection rate. Furthermore, a high CCI score was significantly associated with infection risk (*p* < 0.01). This findings are in line with the current literature, where the negative impact of comorbidities (CCI) on the outcome of spinal surgery has been widely demonstrated [13,34,35,36].

This study had several limitations, first its retrospective nature. Prospective studies are needed to assess the roles of sarcopenia and osteopenia and their effects on the outcome of lumbar surgery. Another limitation is that only PLVI and M score were employed to measure the central sarcopenia and bone mass density, although several other methods are available, such as measurement of muscular strength and of physical performance for sarcopenia [37], and DEXA for bone mass density. However, these tests of course cannot be performed retrospectively and results may be altered in patients undergoing spinal surgery, due to neurological symptoms and muscle weakness. Additional concerns may arise regarding the secondary involvement of the psoas muscle in the context of atrophy caused by wasting accompanying chronic low back pain, affecting its ability to act as a systemic indicator of sarcopenia. However, the literature shows that psoas involvement is ancillary, identifying only the paraspinal muscles (particularly the multifidus) as the main subjects of atrophy.

## 5. Conclusions

Our results indicate that a low M score is a risk factor for SSI, while a low PLVI is not. This confirms that osteopenia could have a great impact on spinal surgery outcomes; however, prospective studies are needed to better investigate the role of muscle and bone tissue quality in predicting outcomes in patients undergoing spinal surgery.

## Figures and Tables

**Table 1:** Baseline characteristic differences for high vs. low PLVI groups, for high vs. low M-score groups, and for non-SSI vs. SSI groups.

Characteristics	Total	Low M Score	High M Score	*p* Value	Low PLVI	High PLVI	*p* Value	Non-SSI	SSI	*p* Value
**n**	308	213	95		153	155		282	26	
**Age at surgery (y. mean.** **±SD)**	63.8 ± 6.2	64 ± 6.4	62.3 ± 5.36	0.05 *	65.3 ± 6.38	62.3 ± 5.7	0.012 *	63.6 ± 5.98	65.9 ± 7.96	0.002 *
**Gender (F)**	160	94	38	0.54	112	48	<0.01*	142	18	0.08
**Diabetes mellitus (yes. n)**	28	20	8	0.04 *	14	14	1	4	24	0.27
**Charlson Comorbidity Index (n. mean.** **± SD)**	2.57 ± 3.6	2.55 ± 1.34	2.54 ± 1.64	0.98	2.77 ± 1.58	2.32 ± 1.02	0.015 *	2.49 ± 1.35	3.38 ± 1.9	<0.01 *
**American Society of Anesthesiology score (n. mean.** **± SD)**	2.03 ± 0.6	2.06 ± 0.58	1.91 ± 0.61	0.07	2.06 ± 0.58	2.02 ± 0.6	0.32	2.01 ± 0.56	2.31 ± 0.74	0.07
**Body mass index (n. mean.** **± SD)**	26.5 ± 6.2	26.7 ± 3.6	25.8 ± 3.5	0.07	27 ± 3.5	25.9 ± 3.6	0.85	26.6 ± 3.6	26.5 ± 4.2	0.98
**Smoking (yes. n)**	74	44	30	0.73	28	46	0.016 *	64	10	0.08
**Length of stay (day. mean.** **± SD)**	11.1 ± 12.7	12.3 ± 15.9	8.6 ± 2.17	0.14	12.23 ± 17.1	10.1 ± 5.5	0.57	9.78 ± 4.8	25.4 ± 38.3	0.27
**Operative time (min. mean** **± SD)**	193.3 ± 59	190 ± 180	204 ± 190	0.06	185.1 ± 62.5	197.4 ± 57.3	0.25	192 ± 59.1	208 ± 58.1	0.24
**PLVI (mean.** **± SD)**	0.71 ± 0.18	0.72 ± 1.19	0.7 ± 0.19	0.5	0.55 ± 0.1	0.88 ± 0.2	<0.01 *	0.71 ± 0.2	0.75 ± 0.62	0.24
**PLVI (low n)**	153	110	43	0.6				141	12	0.7
**M score (mean.** **± SD)**	0 ± 128	−0.47 ± 42.9	1.27 ± 8.1	<0.01 *	0.06 ± 1.02	−0.06 ± 1	0.36	0.03 ± 1.02	−0.20 ± 0.62	0.29
**M score (low n)**	213				135	78	0.6	193	20	0.04 *
**Infection (n. %)**	8.4%	76.9%	22.9%	0.04 *						

Bold and *: the statistically significant results.

**Table 2:** Multivariate linear regression of risk factors for infection. F = female, PVLI = psoas to lumbar vertebral index. Length of stay, age, comorbidity index, ASA score and M score were independent risk factors for infection.

	Estimate	95% CI Lower	95% CI Upper	*p* Value
**Age at surgery**	−0.00254	−0.41195	−0.03125	**0.02**
**Gender (*F*)**	0.03353	−0.05728	0.12435	0.47
**Length of stay**	0.00617	0.00351	0.00884	**<0.001**
**Diabetes mellitus (*yes*)**	−0.02197	−0.17905	0.13531	0.78
**Charlson Comorbidity Index**	0.06126	0.02435	0.09818	**0.001**
**American Society of Anesthesiology score**	−0.08545	−0.16355	−000734	**0.03**
**Body mass index**	0.01468	0.00317	0.02619	**0.01**
**Smoking (*yes*)**	−0.03029	−0.11970	0.05912	0.5
**PLVI**	0.30726	−0.06288	0.67739	0.10
**M score**	−0.16560	−0.30195	−0.02925	**0.01**

Bold: the statistically significant results.

## Data Availability

Not applicable.
